# Measles Epidemiology and Coverage of Immunization Against Measles in the Autonomous Province of Vojvodina, Serbia: Local Trends in a Regional Context

**DOI:** 10.3390/vaccines13070711

**Published:** 2025-06-30

**Authors:** Mioljub Ristić, Svetlana Ilić, Smiljana Rajčević, Mirjana Štrbac, Snežana Medić, Tatjana Pustahija, Vladimir Vuković, Marko Koprivica, Gorana Dragovac, Vladimir Petrović

**Affiliations:** 1 Institute of Public Health of Vojvodina, 21000 Novi Sad, Serbia; svetlana.ilic@izjzv.org.rs (S.I.); smiljana.rajcevic@mf.uns.ac.rs (S.R.); mirjana.strbac@izjzv.org.rs (M.Š.); snezana.medic@mf.uns.ac.rs (S.M.); tatjana.pustahija@mf.uns.ac.rs (T.P.); marko.koprivica@izjzv.org.rs (M.K.); gorana.dragovac@mf.uns.ac.rs (G.D.); vladimir.petrovic@mf.uns.ac.rs (V.P.); 2 Department of Epidemiology, Faculty of Medicine, University of Novi Sad, 21000 Novi Sad, Serbia

**Keywords:** measles, vaccination coverage, immunization programs, outbreaks, epidemiology, herd immunity, Serbia, AP Vojvodina

## Abstract

Background: Despite ongoing global elimination efforts, measles remains a persistent public health threat. Methods: This retrospective observational study examines trends in crude measles incidence and vaccination coverage from 1948 to 2024 in the northern region of Serbia—Autonomous Province of Vojvodina (AP Vojvodina)—which accounts for 26.9% of the national population. This study further explores measles vaccination coverage across the province’s seven districts, along with the number of reported measles cases, age distribution, and vaccination status of affected individuals from 2000 to 2024. Data were obtained from official annual immunization records maintained by public health institutions within the framework of Serbia’s national mandatory immunization program. Results: A notable resurgence of measles occurred in Serbia during 2017–2018, following a decline in vaccination coverage. In AP Vojvodina, outbreaks were recorded in 2007, 2014–2015, and 2017–2018, predominantly affecting unvaccinated children and adults aged 20–39 years. Since 2019, the measles incidence has significantly declined. During the 2018 outbreak, the highest incidence was observed among children aged 1–4 years (40.6 per 100,000), followed by infants under 1 year (17.3 per 100,000) and adults aged 20–39 years (12.5 per 100,000). An analysis of the data from 2000 to 2024 revealed substantial age- and dose-related differences in measles incidence, particularly among unvaccinated individuals, those who had received one or two doses of a measles-containing vaccine (MCV), and those with unknown vaccination status. During the 2017–2018 epidemic, unvaccinated children under 1 year and those aged 1–4 years were the most affected. A marked increase in cases among single-dose recipients was noted in 2018, especially in adults aged 20–39 years (9.5%) and those ≥40 years (13.5%). A considerable proportion of measles cases in these age groups had unknown vaccination status: 33.1% among individuals aged 20–39 years and 18.2% among those aged ≥ 40 years. Epidemiological investigation linked the 2007 and 2014–2015 outbreaks in AP Vojvodina to importations from Bosnia and Herzegovina. No specific source was identified for the 2017–2018 outbreak, suggesting possible endemic transmission. Conclusions: These findings underscore the impact of fluctuating vaccination coverage on measles resurgence. Sustaining high two-dose MCV coverage, strengthening routine immunization programs, enhancing surveillance systems, and ensuring timely outbreak preparedness are critical measures for achieving effective measles control.

## 1. Introduction

Measles continues to pose a significant public health challenge despite decades of global vaccination initiatives. Effective and timely surveillance is crucial for the early detection of outbreaks, monitoring of transmission dynamics, and accurate estimation of disease burden. However, reported case counts often substantially underestimate the true incidence due to factors such as underreporting, limited healthcare-seeking behavior, and delays in data reporting—typically ranging from one to two months [1,2].

In 2024, a total of 35,212 measles cases were reported across the European Union/European Economic Area (EU/EEA), representing a more than tenfold increase compared to the 3973 cases reported in 2023. After a period of atypically low incidence from 2020 to 2022, largely attributed to the COVID-19 pandemic and related public health measures, measles activity began to rise again in 2023. A seasonal transmission pattern reemerged in 2024, marking a return to the epidemiological behavior observed in pre-pandemic years.

Cases were reported across all age groups, with the highest incidence in infants under one year of age, followed by children aged 1–4 years. Notably, individuals aged 15 years and older accounted for 26% of all reported cases, and in some countries, adults over the age of 30 represented the majority of cases (ranging from 28% to 53%). Among the 23 measles-related deaths recorded in 2024—22 of which occurred in Romania—14 were in children under the age of five [3].

The World Health Organization (WHO) recommends maintaining at least 95% coverage with two doses of a measles-containing vaccine (MCV I and MCV II) to achieve herd immunity and prevent outbreaks [4]. Nevertheless, several European countries—particularly in Southeastern and Central Europe—have experienced periodic measles outbreaks over the past two decades, largely as a result of fluctuating vaccination coverage [5].

The Global Vaccine Action Plan, endorsed in 2012, set ambitious targets to eliminate measles in four WHO regions by 2015. These goals included achieving at least 90% national coverage with MCV I, reducing the measles incidence to fewer than five cases per million population, and decreasing measles-related mortality by 95% compared to 2000 levels. By 2012, global measles incidence had declined by 72%, and measles-related deaths by 75%. Moreover, 19 countries in the WHO European and Western Pacific regions have verified the interruption of endemic measles transmission [6].

Despite these achievements, measles elimination remains elusive. In 2023, a total of 60,860 measles cases and 13 deaths were reported in 41 countries of the WHO European Region, with six countries accounting for 95% of all cases. The majority of infections occurred in children under 10 years of age, and genotype D8 was the predominant strain identified [7]. These trends highlight the ongoing need for strengthened political commitment, increased vaccination coverage, enhanced surveillance systems, and improved outbreak response capacity.

Measles has been a notifiable disease in the Autonomous Province of Vojvodina (AP Vojvodina), Serbia—which accounts for 26.9% of the total Serbian population—since 1948. Mandatory measles immunization with a single monovalent vaccine dose was introduced in 1971, targeting all children aged 12–15 months. In 1986, the combined measles–mumps (MM) vaccine became available, and in 1993, it was replaced with the combined measles–mumps–rubella (MMR) vaccine. A two-dose MMR schedule was introduced in 1994, administered at 12–15 months and at 12 years of age. In 2006, the second dose was rescheduled to preschool age (6–7 years) [8].

The objective of this present study was to analyze measles incidence and vaccination coverage trends in AP Vojvodina, Northern Province of Serbia, from 2000 to 2024. Additionally, this study aimed to evaluate the impact of fluctuating vaccination coverage on the occurrence of measles outbreaks in a region with a documented history of periodic epidemics. By leveraging epidemiological and immunization data, this study seeks to inform and support public health strategies to maintain high and equitable vaccine coverage and to prevent future outbreaks.

## 2. Materials and Methods

### 2.1. Study Design and Data Sources

This retrospective observational study analyzed all officially reported measles cases in AP Vojvodina. As previously described, measles cases were classified based on the results of PCR or serological testing [1,4,8]. It aimed to assess crude measles incidence and MCV coverage from 1948 to 2024. Considering that MMR became available in 1993, for the period 2000–2024, the dataset included the number of measles cases and the coverage rates for both the first (MMR I) and second (MMR II) vaccine doses across the seven districts of AP Vojvodina. Additionally, data on age-specific measles incidence and vaccination status by age group in AP Vojvodina were collected.

### 2.2. Data Collection and Variables

The following variables were included in the analysis:

- Annual measles incidence rate: Number of reported cases per 100,000 population per year in AP Vojvodina.
- Age-specific measles incidence rate: Number of reported cases per 100,000 population within specific age groups in AP Vojvodina.
- MCV I (MMR I) coverage: Annual percentage of the target population (at 12–15 months) receiving the first dose of vaccine against measles.
- MCV II (MMR II) coverage: Annual percentage of the target population (at 12 years in the period 1994–2005; at 6–7 years in the period 2006–2024) receiving the second dose of vaccine against measles.

Vaccination coverage in Serbia is monitored using the administrative method, calculated as the proportion of vaccine doses administered to the eligible birth cohorts from the preceding year [8,9]. Individuals without any record of measles immunization were considered unvaccinated. Those with no information or with explicitly missing data were classified as “unknown.” Individuals who received one or two documented doses of MMR were considered vaccinated with either MMR I or MMR II, respectively.

### 2.3. Data Analysis

Descriptive statistical methods were used to assess trends in measles incidence and vaccination coverage throughout the study period. Time-series graphs were constructed to visualize trends, fluctuations, and possible outbreak patterns. All annual data were officially reported to the Center for Disease Control and Prevention at the Institute of Public Health of Vojvodina, Novi Sad, as part of routine communicable disease surveillance in the province. Surveillance was conducted in collaboration with the other district Institutes of Public Health, located in Subotica, Sombor, Pančevo, Sremska Mitrovica, Kikinda, and Zrenjanin.

### 2.4. Ethical Considerations

In accordance with national legislation and institutional policies in Serbia, retrospective analyses of anonymized data do not require approval by an Ethics Committee. The authors were not involved in the treatment of the included patients and did not have access to information that could identify individual participants during and after data collection.

## 3. Results

### 3.1. Trends in Measles Incidence and MCV Coverage in AP Vojvodina, 1948–2024

**Figure 1:**
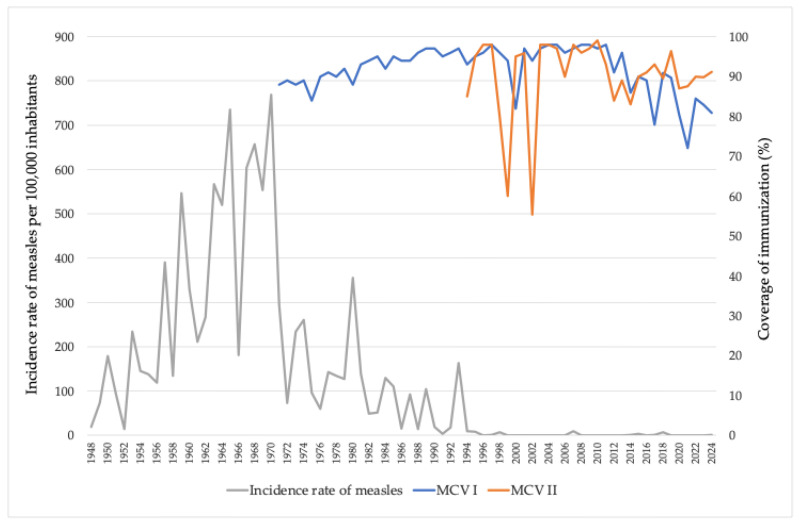
Trends in measles incidence and MCV vaccination coverage in AP Vojvodina, 1948–2024. Legend: MCV—measles-containing vaccine.

The measles incidence rate in AP Vojvodina demonstrated marked variability prior to the introduction of the MCV, with epidemic peaks observed in several years, most notably in 1959 (546.7 per 100,000), 1963 (566.8), 1965 (735.5), 1967 (604.0), 1968 (656.7), and 1970 (768.8). During the first year of immunization against measles (1971), vaccination coverage reached 88.0%. The introduction of the vaccine was followed by a substantial decline in measles incidence, although intermittent outbreaks continued throughout the 1970s and 1980s. In the early 1990s, high MCV I coverage was maintained (≥95% in 1991–1993), and MCV II was introduced in 1994, initially with 85% coverage. From 1995 to 2015, coverage for both MCV I and MCV II generally exceeded 90%, which corresponded with a sustained decrease in measles incidence, including several years with zero reported cases. Between 2001 and 2006, there were no measles cases in AP Vojvodina. A notable resurgence occurred in 2007 (9.8/100,000) despite high vaccination coverage, and again in 2015 (4.1/100,000), 2017 (1.3/100,000), 2018 (7.7/100,000), and 2024 (1.1/100,000). These sporadic outbreaks often coincided with slight declines in MCV I or MCV II coverage. The most pronounced recent decline in first-dose coverage was observed in 2021 (72.1%), likely influenced by disruptions during the COVID-19 pandemic. Although a gradual recovery in coverage was observed from 2022 onward, incidence in 2024 slightly increased to 1.1 per 100,000 (Figure 1).

### 3.2. MMR Vaccination Coverage and Measles Incidence Across the Districts of AP Vojvodina, 2000–2024

Between 2000 and 2024, trends in MMR (measles–mumps–rubella vaccine, introduced in 1993) vaccination coverage and measles incidence exhibited notable variation across the seven administrative districts of AP Vojvodina. While MMR I coverage rates were generally high in the early 2000s across districts of AP Vojvodina, a decline became apparent beginning in 2012, particularly in the South Bačka and West Bačka districts. MMR II coverage was lower in the early years of the study period compared to subsequent years.

In South Bačka, MMR I coverage dropped markedly from 2012 to 2017, reaching a low of 61.6% in 2017. This district also recorded the highest measles burden during the 2007–2018 period, including 148 cases in 2007 and 69 in 2018. In West Bačka, a notable decline in MMR I uptake was observed in 2024, with coverage falling to 68.4%. In contrast, districts such as North Banat, Central Banat, South Banat, and Srem maintained relatively stable coverage over the 25-year period. The average MMR I coverage in these districts ranged from 91.9% in Central Banat to 96.0% in North Banat, while MMR II coverage generally remained between 91.8% and 94.4%.

Isolated measles cases were sporadically reported in Central Banat (2007, 2009, 2015, and 2018) and South Banat (2000, 2014, 2017, 2018, and 2024), with the largest cluster in South Banat occurring in 2018 (20 cases). North Bačka experienced resurgences in 2015 (eight cases) and 2018 (three cases), despite maintaining vaccination coverage above 90% throughout this period.

**Figure 2:**
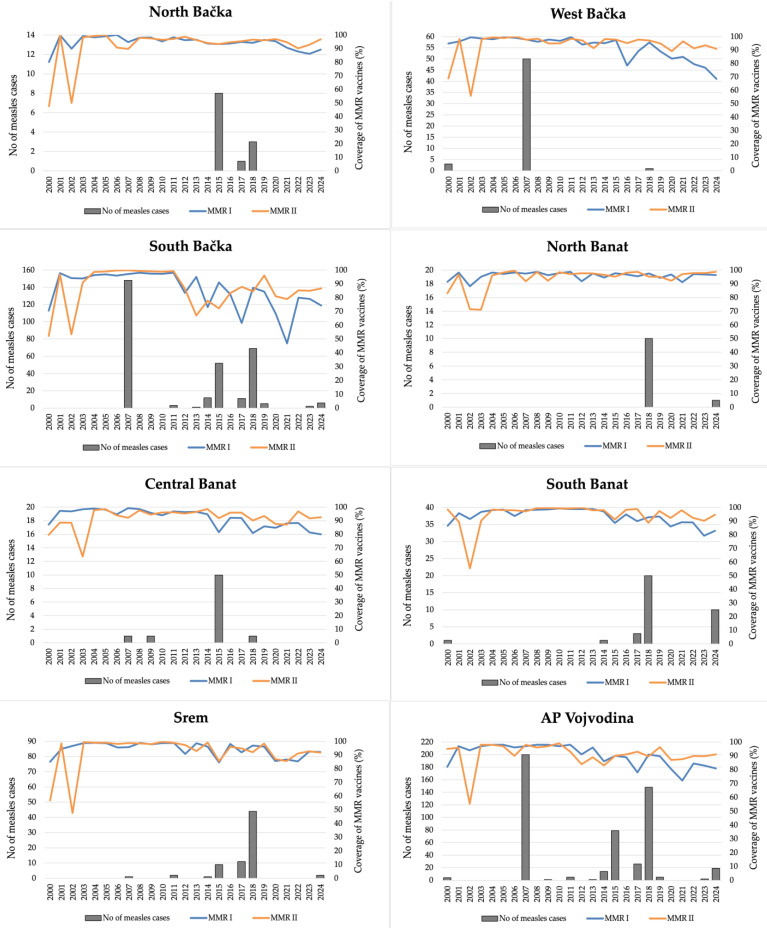
Coverage of MMR vaccination and measles cases across districts of AP Vojvodina, 2000–2024.

At the level of AP Vojvodina, MMR I coverage demonstrated a consistent downward trend, decreasing from 98.0% in 2004 to 72.1% in 2021. In comparison, MMR II coverage was more variable, ranging from 55.4% in 2002 to a peak of 99.0% in 2010. The most significant outbreaks during the study period were recorded in 2007 (200 cases) and 2018 (148 cases), highlighting the potential consequences of declining vaccination coverage (Figure 2).

### 3.3. Measles Cases by Age Groups in AP Vojvodina, 2000–2024

**Figure 3:**
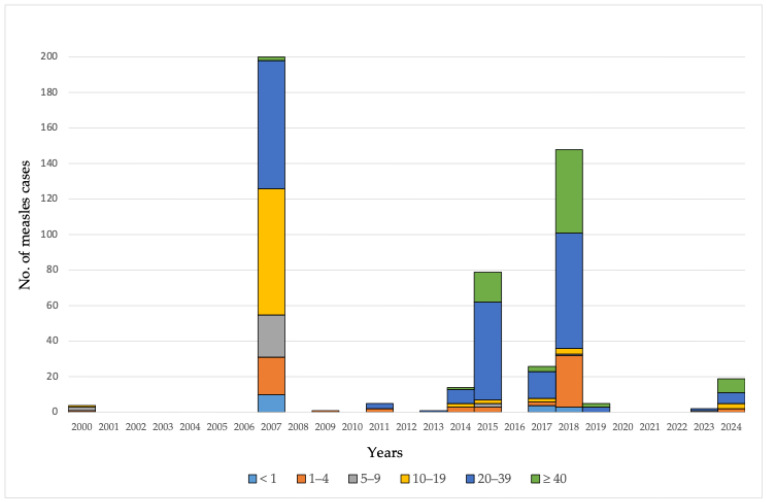
Measles cases by age groups in AP Vojvodina, 2000–2024.

From 2000 to 2024, the incidence rate of measles in AP Vojvodina demonstrated fluctuating trends across different age groups. In 2000, four cases were reported, all among children aged 1–19 years. No cases were recorded between 2001 and 2006. A major outbreak occurred in 2007, with 200 reported cases distributed across all age groups. The highest number of measles cases were observed among individuals aged 10–19 years (71 cases, 35.5%) and 20–39 years (72 cases, 36%). In the following years, measles cases were sporadic. A single case was reported in 2009 (child aged 1–4 years), and five cases were recorded in 2011. Between 2012 and 2015, cases were rare, with only one case reported in 2013 (20–39 years) and 14 cases in 2014 across various age groups. A notable resurgence occurred in 2015, with 79 cases, predominantly among individuals aged 20–39 years (55 cases, 69.6%), followed by 17 cases in those aged ≥ 40 years. After a decline in 2016, a total of 26 cases were reported in 2017, mainly among adults aged 20–39 years (fifteen cases, 57.7%) and infants (four cases). In 2018, another significant outbreak resulted in 148 cases, with the largest burden observed in the 20–39 age group (65 cases, 43.9%) and among individuals aged ≥ 40 years (47 cases, 31.8%). From 2019 onward, the incidence of measles declined markedly. In 2019, five cases were reported—three in the 20–39 age group and two in those aged ≥ 40 years—while no cases were recorded between 2020 and 2022. In 2023, two cases were registered: one in a child aged 5–9 years and one in an adult aged 20–39 years, and in 2024, there were 19 measles cases, mostly (14/19; 73.7%) among persons aged ≥ 20 years (Figure 3).

### 3.4. Incidence Rates of Measles by Age Groups in AP Vojvodina, 2000–2024

**Figure 4:**
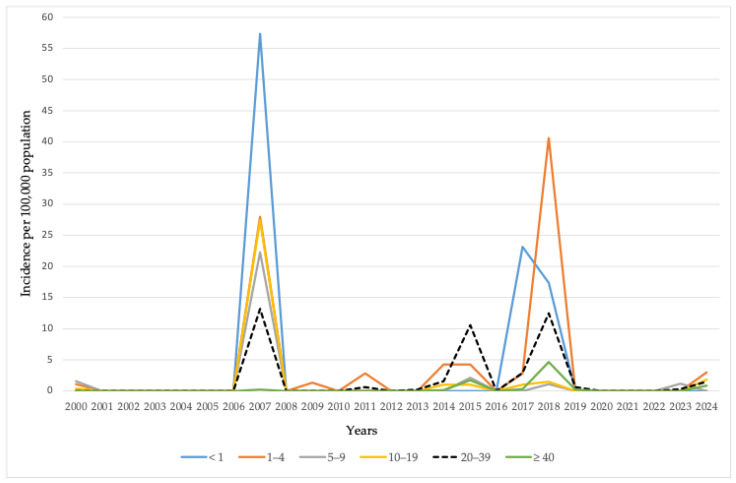
Incidence rates of measles by age groups in AP Vojvodina, 2000–2024.

Measles incidence in AP Vojvodina has varied across age groups over the years, reflecting intermittent transmission and age-specific vulnerability. In 2000, cases were primarily reported among children aged 1–4 years (1.0 per 100,000) and 5–9 years (1.5 per 100,000). A marked increase was observed in 2007, with the highest incidence recorded in infants under 1 year of age (57.4 per 100,000) and children aged 1–4 years (27.9 per 100,000), while elevated rates were also noted across older age groups. Incidence declined significantly in 2009, with only one reported case in the 1–4 age group (1.3 per 100,000) and no cases in other age categories. In 2011, a modest increase was observed among children aged 1–4 years (2.8 per 100,000) and adults aged 20–39 years (0.6 per 100,000). A resurgence of measles occurred in 2014, with the highest incidence in children aged 1–4 years (4.2 per 100,000), accompanied by moderate rates in older age groups. The situation intensified in 2015, with a notable rise in incidence among adults aged 20–39 years (10.6 per 100,000), alongside lower but still significant rates in younger children. In 2017, the overall incidence declined but remained elevated in infants under 1 year (23.1 per 100,000) and adults aged 20–39 years (2.9 per 100,000). The 2018 outbreak yielded the highest incidence in children aged 1–4 years (40.6 per 100,000), followed by infants under 1 year (17.3 per 100,000) and adults aged 20–39 years (12.5 per 100,000). By 2019, measles incidence had decreased substantially, with sporadic cases confined mostly to adults. In 2023, a low incidence was recorded, primarily in children aged 5–9 years (1.2 per 100,000), with minimal case occurrence in older age groups. During 2024, the highest incidence rate of measles (3.0 per 100,000) was recorded among children aged 1–4 years, while the incidence rates in other age groups did not exceed 1.7 per 100,000 (Figure 4).

### 3.5. Measles Cases by Age Group and Vaccination Status Against Measles in AP Vojvodina, 2000–2024

**Figure 5:**
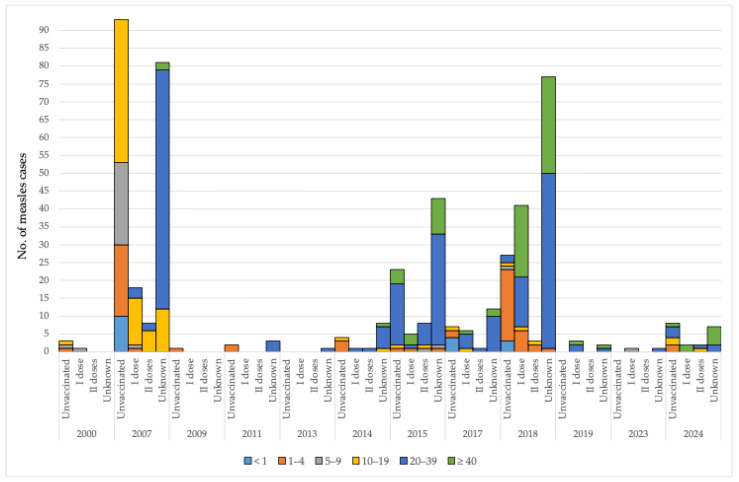
Measles cases by age groups and vaccination status against measles in AP Vojvodina, 2000–2024.

Data on measles cases in AP Vojvodina from 2000 to 2024 reveal substantial variation across age groups and vaccination statuses, distinguishing among unvaccinated individuals, those who received one or two doses of the MCV, and individuals with unknown vaccination status. In 2000, the majority of reported cases occurred among unvaccinated children in younger age groups. One case was recorded in an individual who had received a single vaccine dose, while no cases were reported among those with two doses or unknown vaccination status. A pronounced increase in measles incidence was observed in 2007, predominantly among unvaccinated individuals, particularly in the 10–19 age group (40 cases, 20%), followed by children aged 5–9 years (23 cases, 11.5%) and 1–4 years (20 cases, 10%). That same year, cases were also reported among individuals who had received one vaccine dose, especially in the 10–19 age group (13 cases, 6.5%), as well as among individuals with unknown vaccination status, notably in the 20–39 age group (67 cases, 33.5%). Between 2009 and 2013, measles cases were sporadic and limited to unvaccinated children and individuals with unknown vaccination status. In 2014, both vaccinated and unvaccinated individuals were affected, with six cases (42.9%) in the 20–39 age group classified as having unknown vaccination status. The 2015 resurgence was marked by an increased number of cases, particularly among unvaccinated individuals aged 1–4 years, 10–19 years, and ≥20 years. A considerable rise in cases with unknown vaccination status was also noted, especially among adults aged 20–39 years (31 cases, 39.2%) and those aged ≥ 40 years (10 cases, 12.7%). During the 2017–2018 period, unvaccinated children under 1 year and those aged 1–4 years remained the most affected groups. A notable increase in cases among single-dose recipients occurred in 2018, particularly among adults aged 20–39 years (14 cases, 9.5%) and ≥40 years (20 cases, 13.5%). Additionally, there was a high number of cases with unknown vaccination status in these age groups—49 cases (33.1%) in 20–39-year-olds and 27 cases (18.2%) in individuals aged ≥ 40 years. From 2019 to 2023, only a few measles cases were reported, primarily among individuals who had received only a single dose of the vaccine or whose vaccination status was unknown. During the last observed year, there were eight (42.1%) cases involving unvaccinated individuals, seven (36.8%) cases with unknown MCV status, and two cases with one or two doses of MCV. Most unvaccinated cases and cases with unknown MCV were among persons aged ≥ 20 years (Figure 5).

## 4. Discussion

### 4.1. Regional Measles Epidemiology and Vaccination Coverage

Based on recent WHO data, the measles incidence in Serbia and neighboring countries remained relatively low during 2000–2023, but intermittent outbreaks occurred, often linked to declines in vaccination coverage [5]. A significant resurgence between 2017 and 2019 highlighted the risks of suboptimal immunization. MCV I and MCV II coverage varies across countries neighboring Serbia, reflecting ongoing challenges to herd immunity [5,10,11,12,13,14,15,16,17,18,19,20,21,22,23,24,25,26,27]. Romania reported the highest notification rate in 2024 among Serbia’s neighbors, accounting for approximately 87% of EU/EEA cases [3].

Countries like Hungary, maintaining high coverage above 95%, sustained near-zero incidence, demonstrating effective immunization programs [5]. Consistent with this, our findings demonstrate that districts of AP Vojvodina with sustained high MCV coverage experienced fewer measles cases compared to the South Bačka district. In contrast, countries with fluctuating or low coverage against measles such as Montenegro, Romania, and Bosnia and Herzegovina faced recurrent large outbreaks and an expanding susceptible population. Delayed outbreaks after coverage declines occurred in Albania and North Macedonia, while Serbia’s major 2017–2018 outbreak coincided with prior vaccination rate reductions [5].

These patterns emphasize the long-term public health impact of reduced vaccine uptake, where immunity gaps may lead to delayed epidemic surges [5]. Strong surveillance and rapid outbreak response complement high vaccine coverage, as seen in Hungary’s experience with imported cases [16,17]. Despite moderate coverage, countries including Bulgaria, Croatia, and Serbia experience outbreaks due to subnational heterogeneities and vaccine hesitancy [12,13,14,15,24,25,26,27].

### 4.2. Measles Notification and Introduction of Immunization

Mandatory measles notification systems were established in Serbia and most neighboring countries by the mid-20th century [10,11,12,13,14,15,16,17,18,19,20,21,22,23,24,25,26,27]. Measles immunization programs were introduced between 1968 and 1979, coinciding with the WHO’s Expanded Program on Immunization (EPI) rollout [28]. Despite early vaccination efforts, large outbreaks have persisted, indicating ongoing immunity gaps and challenges in sustaining high coverage. Most countries use passive surveillance systems and apply EU or WHO case definitions. While passive surveillance is cost-effective and widespread, it is limited by underreporting and delayed outbreak detection. Standardized case definitions aid data comparability, but differences in implementation can affect sensitivity and timeliness [10,11,12,13,14,15,16,17,18,19,20,21,22,23,24,25,26,27].

### 4.3. Measles Outbreaks in Serbia and Neighboring Countries Between 2000 and 2023

Between 2014 and 2019, nearly all countries in the region experienced significant measles outbreaks, with Romania and North Macedonia reporting multiple waves [20,21]. In Romania, measles transmission has continued into 2023, with circulating genotypes B3 and D8, which are consistent with those identified elsewhere in Europe [21,22]. Serbia’s large 2017–2018 outbreak was mainly caused by genotype B3, similar to neighboring countries, highlighting the need for regional coordination [10,13,14,18,20,21,22,23].

The 2009–2011 outbreak in Bulgaria was the largest in the WHO European Region at that time, resulting in over 24,000 cases and 24 deaths, predominantly among Roma children under 15 [5,12]. A similar pattern was observed in AP Vojvodina in 2007 [29]. Hungary maintained high vaccination coverage, reporting only sporadic cases from 2000 to 2023, with the 2017 outbreak involving healthcare workers exposed to unvaccinated Romanian children; genotype B3 was confirmed [16,17].

Montenegro, North Macedonia, and Romania showed different epidemiological trends. Montenegro had the lowest vaccination coverage but a lower measles incidence than the other two, possibly due to low population density limiting transmission [5,18]. North Macedonia reported outbreaks in 2010–2011 and 2019, with low vaccination rates and fatal cases, and genotype D4 circulating from the UK to several countries including Serbia [19,20,30]. Romania experienced a major outbreak from 2016 to 2019 with over 21,000 cases, mostly unvaccinated young children, and several deaths [5,21,22].

Despite suboptimal coverage in Serbia, no measles importations from Romania have been documented, potentially due to relatively higher immunization rates in border districts—North and South Banat [31]. In contrast, Hungary experienced adult measles cases imported from Romania despite near-universal vaccination [16,17,21].

### 4.4. Measles Outbreaks in AP Vojvodina Between 2000 and 2024

Regarding the epidemiological situation of measles in AP Vojvodina, the number of measles cases has fluctuated significantly over time, with notable outbreaks occurring in 2007, 2014–2015, and 2017–2018. Interestingly, the majority of cases in the 2007 outbreak were among children while in 2014–2015 they were predominantly among children and students, with unvaccinated individuals representing the largest share of those affected in both periods [8,29]. In contrast, the 2017–2018 epidemic saw a significant proportion of cases in individuals over 20 years of age, accompanied by an increase in cases among those who had received only one dose of the MCV [26,31].

The importation of measles cases in AP Vojvodina was traced to Bosnia and Herzegovina in both the 2007 and 2014–2015 outbreaks while in the 2017–2018 outbreak, the origin was unknown; however, the first cases were reported in Kosovo and Metohija [26,32].

Similar to risk assessments conducted in Romania [21], a seroepidemiological study performed in AP Vojvodina prior to the 2017–2018 outbreak revealed that 56% of children aged 12–24 months and 19% of individuals aged 20–39 years were seronegative for measles. In line with these findings, during the mentioned outbreak, 91 out of 177 cases (51.4%) were in the 20–39 age group, and the mean age of all measles cases was 29 years. Furthermore, more than half of the reported cases in AP Vojvodina during the 2017–2018 outbreak (97/177; 54.8%) had received only one dose of MCV [31]. In response to this outbreak, a series of timely interventions were implemented in AP Vojvodina, including the immediate vaccination of all healthcare workers employed in facilities with a high risk of measles-related complications, as well as the vaccination of all parents or guardians accompanying hospitalized children. In addition, measles cases were diagnosed within one day, and a catch-up immunization campaign was launched targeting all individuals who had been in contact with confirmed measles cases within a three-day window. These measures likely contributed to the fact that only 3% of the total measles cases recorded in Serbia during this epidemic occurred in AP Vojvodina [8,33,34]. This coordinated approach may serve as a useful model for other regions.

### 4.5. Factors Potentially Associated with the Decline in Measles Immunization Coverage

Given the historical pattern of measles transmission, Serbia is considered an endemic country for measles [25,27]. Due to persistent challenges in maintaining sufficiently high coverage rates for MCV, the goal of measles elimination has been postponed to 2030, as opposed to the original target set for 2010 [27,35]. Even prior to the COVID-19 pandemic, coverage rates for both MCV I and MCV II in Serbia were already below the WHO-recommended thresholds [27].

Excluding the negative impact of the COVID-19 pandemic on immunization services, several factors contributed to declining MCV coverage in the region. These include demographic changes and uncontrolled population movements, notably in Albania (1990–2010) [10], as well as rising vaccine hesitancy due to perceived low measles risk, mistrust in experts, misinformation—especially in urban areas—and safety concerns. Anti-vaccination movements have further increased outbreak risks [11,14,18,22,23,36,37,38]. In Serbia, delays in the timely availability of the MMR vaccine were documented between 2012 and 2016 [31], which is unacceptable for a country with mandatory childhood immunization and may undermine public confidence in the immunization system. Moreover, despite the limited availability of MMR doses—particularly during the early years of the study period—priority was given to administering the MMR I at the expense of the MMR II, resulting in higher coverage rates for MMR I.

Barriers to achieving target MCV coverage extend beyond the general population to healthcare professionals, where lack of confidence can cause false contraindications and vaccination delays, undermining public trust [15]. Parental decisions are influenced by trust in experts and vaccines, perceived measles severity, responsibility for child and community health, and social pressures [36]. Notably, regarding our previous experiences, a dramatic increase in MCV coverage is often observed during measles outbreaks, frequently exceeding coverage levels recorded in the pre-epidemic period [8,33,34].

Although our findings indicate that some of the observed countries experienced multiple measles outbreaks between 2000 and 2023 despite relatively high reported MCV coverage, this paradox can plausibly be attributed to limitations inherent in administrative methods used to estimate immunization coverage. In particular, overestimations may occur when denominators exclude unregistered or underserved populations, such as Roma communities, thereby obscuring the actual levels of immunization. For example, despite high national-level MCV coverage reported in Bulgaria, the country experienced the largest measles outbreak in Europe during the first decade of the 21st century, primarily due to such underrecognized pockets of susceptibility [12]. A similar situation was documented in AP Vojvodina in 2007 [29]. Furthermore, the ongoing war in Ukraine and the increasing number of refugees represent an additional risk for the emergence of new measles outbreaks across Europe. In tourist regions that host large numbers of visitors and seasonal workers from various parts of the world—such as Split-Dalmatia County and Montenegro—the identification of susceptible population pockets and the implementation of catch-up vaccination strategies may once again become critical [13,18].

Finally, it is well established that measles can be eliminated—and potentially eradicated—due to its favorable virological and epidemiological features: humans are the only reservoir, there is no prolonged viral carriage or significant antigenic variation, and vaccines protect against all strains [15,22,39]. The vaccines are low-cost, highly effective, provide long-term immunity, and have an excellent safety profile. However, achieving elimination requires stronger and more comprehensive efforts, as mandatory vaccination alone does not guarantee high coverage [15,40].

This study has several limitations that should be considered when interpreting the results. First, as a retrospective observational study relying on officially reported measles cases, it is subject to potential underreporting and reporting bias, which may affect the accuracy of the incidence estimates. Second, vaccination coverage data were obtained using the administrative method, which calculates coverage based on the number of vaccine doses administered relative to the eligible birth cohorts. This approach may overestimate coverage due to inaccuracies in denominator data or incomplete records. Third, individuals with missing or unknown vaccination status were classified separately, but the possibility of misclassification remains, which could influence the analysis of vaccination impact. Additionally, this study is limited to data from AP Vojvodina and may not be fully generalizable to other regions of Serbia or countries with different immunization practices. Despite the above-mentioned limitations, we believe that the main findings of our study remain valid.

## 5. Conclusions

Addressing declining immunization trends through targeted public health interventions remains essential for long-term measles control in our territory. We found that during the 2007 outbreak, most cases occurred among children, whereas in 2014–2015, they were mainly reported among children and students, with unvaccinated individuals comprising the largest proportion in both periods. In contrast, the 2017–2018 epidemic involved a substantial number of cases in people older than 20 years, along with an increase in cases among those who had received only a single dose of the MCV. These results emphasize the urgent need for strengthening measles control efforts, including enhanced surveillance systems (potentially integrating active components), ensuring high two-dose vaccination coverage, and improving outbreak preparedness. Experiences from measles outbreaks in AP Vojvodina can help strengthen control measures in settings with low immunization coverage, both in our country and in other regions facing similar public health challenges.

## Data Availability

The data that support the findings of this study are available from the corresponding author upon reasonable request.
